# A Harmonised Approach to Curating Research-Ready Datasets for Asthma, Chronic Obstructive Pulmonary Disease (COPD) and Interstitial Lung Disease (ILD) in England, Wales and Scotland Using Clinical Practice Research Datalink (CPRD), Secure Anonymised Information Linkage (SAIL) Databank and DataLoch

**DOI:** 10.2147/CLEP.S437937

**Published:** 2024-04-04

**Authors:** Sara Hatam, Sean Timothy Scully, Sarah Cook, Hywel T Evans, Alastair Hume, Constantinos Kallis, Ian Farr, Chris Orton, Aziz Sheikh, Jennifer K Quint

**Affiliations:** 1 Usher Institute, The University of Edinburgh, Edinburgh, UK; 2 Population Data Science, Swansea University Medical School, Swansea, UK; 3 School of Public Health, Imperial College London, London, UK; 4 EPCC, The University of Edinburgh, Edinburgh, UK

**Keywords:** COPD, asthma, ILD, HER, harmonisation, data curation

## Abstract

**Background:**

Electronic healthcare records (EHRs) are an important resource for health research that can be used to improve patient outcomes in chronic respiratory diseases. However, consistent approaches in the analysis of these datasets are needed for coherent messaging, and when undertaking comparative studies across different populations.

**Methods and Results:**

We developed a harmonised curation approach to generate comparable patient cohorts for asthma, chronic obstructive pulmonary disease (COPD) and interstitial lung disease (ILD) using datasets from within Clinical Practice Research Datalink (CPRD; for England), Secure Anonymised Information Linkage (SAIL; for Wales) and DataLoch (for Scotland) by defining commonly derived variables consistently between the datasets. By working in parallel on the curation methodology used for CPRD, SAIL and DataLoch for asthma, COPD and ILD, we were able to highlight key differences in coding and recording between the databases and identify solutions to enable valid comparisons.

**Conclusion:**

Codelists and metadata generated have been made available to help re-create the asthma, COPD and ILD cohorts in CPRD, SAIL and DataLoch for different time periods, and provide a starting point for the curation of respiratory datasets in other EHR databases, expediting further comparable respiratory research.

## Introduction

Asthma, chronic obstructive pulmonary disease (COPD) and interstitial lung disease (ILD) are chronic respiratory diseases associated with substantial disability and mortality worldwide.^1^

Asthma, a chronic inflammatory respiratory disease, associated with airway inflammation and hyper-responsiveness and is characterised by cough, wheeze and chest tightness. It is common across Europe, with ~30 million diagnosed cases among children and adults aged <45 years. In the United Kingdom (UK), over 5.4 million people have asthma,^2^ accounting for over 65,000 hospital admissions and 1000 deaths annually.^3^ In 2016/17, more than 75,000 people spanning all age groups experienced an asthma exacerbation that required hospitalisation.

COPD is a chronic condition characterised by progressive airflow obstruction, which is not completely reversible.^4^,^5^ In 2020/21, the prevalence of COPD in England was estimated at 1.9%, which equated to approximately 1.17 million people.^6^ COPD contributes to nearly 30,000 deaths each year in the UK, corresponding to 5.7% of adult male and 4.0% of adult female deaths, including a substantial number of premature deaths.^7^

ILD encompasses a heterogeneous group of disorders, ranging from conditions that completely resolve without requirement for pharmacological intervention through to fibrotic lung diseases, which inexorably progress to respiratory failure and death despite treatment. ILD is thus an umbrella term used to represent a diverse group of lung conditions with different aetiologies, unpredictable progression and varying survival times. There is a large variation in global prevalence and burden, due in part to varying ontologies and diagnostic accuracy.^8^ Burden is greatest in those fibrotic ILDs, and in particular, those that are progressive.^9^ The most common ILD is idiopathic pulmonary fibrosis (IPF), which has attracted the most research interest in recent years.^8^ This is because not only because it is the most prevalent of the ILDs, but also because it has a universally progressive nature and a poor prognosis. Additionally, the UK has one of the highest incidence of IPF, making research into IPF of particular interest to UK health organisations.^10^

As the digitisation of health systems rapidly matures, there is an accompanying proliferation of research that is now capitalising on this digital ecosystem. EHRs are an increasingly important resource to help improve patient outcomes in chronic respiratory disease.^11^ However, without appropriate data cleaning and curation, the potential of EHRs is limited. There is also now a substantial body of evidence indicating that the lack of harmonisation of definitions and approaches for exposures and covariates can generate very considerable variations in estimates of, for example, disease incidence and prevalence due to misclassification and omission of individuals who should be included.^12^,^13^ Curated datasets that are continuously maintained with commonly used variables would help to ensure consistent definitions between analyses and avoid duplication of effort of data cleaning for every new study.

Our research aims were to improve understanding of the evolving impacts of these diseases on individuals and the associated burden in England, Wales and Scotland for public health planning, and identifying areas for future research and development to improve patient care through creation of harmonised curated datasets for asthma, COPD and ILD in each country using existing EHR databases. Furthermore, we aimed to generate a continuously maintained cohort of all asthma, ILD and COPD patients in each population by combining disease-related data variables across several different linked datasets, in order to describe the following for each disease: (I) common variables (ie age, smoking status, body mass index (BMI), spirometry for COPD only) at diagnosis and (II) cohort description on 31st December 2019. For asthma and COPD, we also described number of people with prescriptions of common asthma and COPD medications.

By parallelising work in England, Wales and Scotland, we aimed to: fill gaps in the understanding of disease burden across these different respiratory diseases in all three countries; improve phenotypic understanding around respiratory conditions and relevant covariates within different EHR datasets; and generate codelists and scripts which can create research-ready datasets that ultimately (with appropriate permissions) can be maintained and re-used by collaborating respiratory researchers, offering the potential for substantial public health benefit.

## Methods Data Sources and Coding Systems

For England, the database used was the Clinical Practice Research Datalink (CPRD) Aurum February 2022 version. The CPRD Aurum database consists of routinely collected, anonymised electronic healthcare record data from general practices (GPs) using the Egton Medical Information Systems (EMIS) system in the UK covering 20% of the English population in February 2022 with 91% of included patients eligible for linkage.^14^ Linked pseudonymised mortality data from the Office for National Statistics (ONS), socioeconomic data from the Index of Multiple Deprivation (IMD), and secondary care data from Hospital Episode Statistics (HES) were provided for this study by CPRD for our patient cohorts.

In Wales, the Secure Anonymised Information Linkage (SAIL) Databank was used; a secure repository of anonymised health and administrative data about the population of Wales that can be linked for research purposes and is accessible in anonymised form via a secure data sharing platform.^15–17^ The Welsh Longitudinal General Practice dataset (WLGP) was used to establish the cohort from primary care records, then linkage was made to the Welsh Demographic Service Dataset (WDSD). WDSD provides demographic information for people registered with a general practice in Wales, including Welsh Index of Multiple Deprivation (WIMD) and Lower Layer Super Output Area (LSOA), containing 88% of the Welsh population (as of the data extract date, 7th of March 2022). Linkage to the Patient Episode Dataset for Wales (PEDW) dataset enabled the use of Welsh hospital inpatient records in SAIL.

For South-East Scotland, the DataLoch service provides de-identified patient-level data with linkage across regional and national routine healthcare datasets.^18^ Primary care datasets cover 88% of Lothian general practices while the secondary care datasets cover the whole Lothian population (approximately 900,000 residents). In May 2023, each cohort was selected using GP Read Code Events associated with registered patients, linked to multiple nationally reported datasets: Scottish Index of Multiple Deprivation (SIMD) from Public Health Scotland (PHS); Scottish Morbidity Records (SMR) datasets from PHS; Prescribing Information System (PIS) for Community Paid prescriptions data (ie prescriptions dispensed by a community pharmacist, appliance supplier or dispensing doctor within the UK and reimbursed by NHS boards through Practitioner Services Division) from PHS; and National Records of Scotland (NRS) death registrations. Additionally, linkage was performed to RespNet – the Lothian secondary care respiratory clinic database – for spirometry values.

Other than population coverage, the main differences between the three datasets are detailed in Table 1.

**Table 1:** Summary of Differences Between CPRD Aurum, SAIL and DataLoch Datasets and Harmonisation Approaches Employed

CPRD Aurum	SAIL Databank	DataLoch	Harmonisation Approach (if Applicable)
Each patient registration given unique identifier (patid)	Each patient given unique Anonymous Linking Field (ALF) identifier	Each patient has unique Community Health Index (CHI) which is pseudonymised on a project-by-project basis	In CPRD, follow-up period was registration start date to earliest of: death date, last practice collection date, and registration end date to avoid counting duplicates. All GP codes outside the registration period were not used in CPRD.
Year of birth only	Week of birth	Full date of birth available but month and year of birth routinely given for research	In CPRD, date of birth was set to 1st July of that year. In SAIL, the Monday of the week of birth is used as the date of birth. When month and year of birth given in DataLoch, date of birth is set to 1st of that month.
Medcodeid used in GP data	5-character Read V2 and V3 codes used in GP data	7-character Scottish Read V2 codes used in GP data	All codelists have medcodeid (CPRD Aurum proprietary medical code identifier) corresponding to the equivalent SNOMED-CT concept and description IDs (with complete coverage) and Read code (only where available) using the CPRD Aurum medical dictionary.
Medications based on GP issued prescriptions all-time	Medications based on GP issued prescriptions all-time	Medications based on GP dispensed prescriptions available from 2009	-
Prodcodeid used for prescriptions	5-character Read Codes Drug and Appliance Dictionary (DAAD) used for prescriptions	Full British National Formulary (BNF) codes used for prescriptions	All codelists have prodcodeid (CPRD Aurum proprietary medicinal product code identifier) corresponding to Dictionary of medicines and devices (dm+d) where available, using the CPRD Aurum product dictionary. dm+d codes were mapped to full BNF codes and Anatomical Therapeutic Chemical (ATC) codes, the latter of which were then mapped to Read codes.
File-based	SQL database	SQL database	Scripts coded in Stata and R in CPRD, and SQL and R in SAIL and DataLoch. R scripts shared across databases where appropriate.
English Index of Multiple Deprivation (IMD) 2019 (1 = least deprived, 5 = most deprived)	Welsh Index of Multiple Deprivation (WIMD) 2019 (1 = most deprived, 5 = least deprived)	Scottish Index of Multiple Deprivation (SIMD) 2020 v2 (1 = most deprived, 5 = least deprived)	IMD 2019 and WIMD 2019/SIMD 2020v2 have opposite scales, therefore results always refer to descriptive terms rather than numeric.

Notably, each patient was given a unique identifier in SAIL and DataLoch, whereas the patient GP registration in CPRD was the unique identifier. This means the same patient in CPRD may have multiple identifiers if they changed GP practice. Therefore, we could only include patients and their events during their registration periods to avoid double counting.

A major challenge in harmonisation was the different coding systems utilised in each database across the GP events and prescriptions. For example, CPRD Aurum only contained GP data from participating EMIS practices that had upgraded to the SNOMED-CT coding system, while SAIL WLGP data were encoded in Read V2 and CTV3 – both of which have been retired by NHS England since 2016 and 2018, respectively,^19^ which may present additional complexities for future studies involving newer codes (such as those for COVID-19), as EMIS and Vision may introduce new Read codes independently from each other.

Similar to SAIL, DataLoch GP data were encoded in Read V2. CPRD provides a “medical dictionary” file to its users with conversions between medcodeid, SNOMED-CT and Read, and this was used to synchronise codes between the coding systems. For medications, CPRD offered users a “product dictionary” file which contained dm+d identifier and British National Formulary (BNF) chapter (where available) for all drugs prescribed in CPRD Aurum, but this did not contain Read codes DAAD, the drug coding system in SAIL WLGP data, nor full BNF codes which were used in DataLoch. While there was a mapping available from dm+d to BNF and Anatomical Therapeutic Chemical code (ATC) on the NHS-hosted Technology Reference Update Distribution (TRUD), we could not find any publicly available conversion tables from Read to either dm+d or BNF. However, an ATC to Read code conversion table existed internally in SAIL, which offered the best approximation and is available on licence (https://factsanddimensions.co.uk/ukhd).

Another key difference in the medications data across the databases was that DataLoch provided dispensed GP prescriptions as part of their core offerings – meaning no visibility of GP prescriptions that were not dispensed – whereas CPRD and SAIL had only GP-issued prescriptions, therefore it was not possible to tell which ones were dispensed.

We defined our study cohorts of individuals with ILD, asthma and COPD in primary care records using the same definitions in CPRD Aurum, SAIL Databank and DataLoch, using validated codelists where possible.^20–22^ All codelists used, including cohort definitions and variables, can be found on our GitHub (https://github.com/NHLI-Respiratory-Epi/Curation-Harmonisation) and on the Health Data Research UK Phenotype Library (https://phenotypes.healthdatagateway.org/).

Inclusion and exclusion criteria

### Common Inclusion Criteria

We included patients with a sex of male or female with at least one valid asthma, COPD or ILD event on or before 31st December 2019 who were alive and permanently registered with a participating GP for any period of time from 1st January 2004. Events were considered valid if they were within patient lifetime and patients were aged at least 35 years old for COPD and at least 40 for ILD.

### Additional Exclusion Criteria

In CPRD, patients were excluded if they did not meet the “acceptable” CPRD quality standards;^23^ were not eligible for linkage to IMD, HES, and ONS; or were in practices with either an unknown region or a region outside England.

Patients were ineligible for inclusion in the DataLoch cohorts if they had an invalid CHI; where date of birth and sex did not match CHI; and where there was significant health activity flagged after death (indicative of a duplicate CHI).

Variable definitions: The creation of variables was based on input from clinicians and from existing evidence on best practice and key findings from leading epidemiological studies. Where applicable, existing validated codelists were utilised.^24^,^25^

### Date of Incidence and Earliest Mention

Date of diagnosis is a key variable for epidemiological research. Incident rates have been noted to be higher in the first year of registration for chronic diseases in primary care records, due to GPs entering prevalent data when a new patient joins the practice which is misconstrued as incident data.^26^ This effect is even stronger in CPRD data due to each patient identifier having a unique registration.

Researchers may be interested in a more reliable date of incidence or just the first mention of disease depending on their study; therefore, we opted to have two diagnosis date variables: date of incidence and earliest mention.

Each asthma, COPD and ILD code were classified as “incident” and/or “prevalent” with clinical input. Codes that could be diagnostic were classified as both incident and prevalent, while disease management codes were set to prevalent only.

Figure S1 shows how date of incidence and earliest mention are selected from the GP events. If the earliest mention was within the first year of registration, then the date of incidence was set to missing to indicate that incidence of disease for the patient likely did not occur during that registration period.

### Date of Death

Date of death was assigned using the ONS mortality register as the primary source for both CPRD and SAIL. In SAIL, the Annual District Death Extract (ADDE), which was derived from the ONS dataset, provided a register of all deaths relating to Welsh residents. In DataLoch, the internally derived date of death was utilised, which incorporated NRS deaths register as the primary source.

In CPRD, if a death record in ONS existed for an individual and date of death was missing, then the ONS death registration date was used. Since the CPRD ONS dataset coverage ended in March 2021, any death dates thereafter were provided from the recommended CPRD death date variable (“cprd_ddate”).

### Follow-Up Start and End Dates

In CPRD, patient follow-up start date was the GP registration start date and the follow-up end date was the earliest of: date of death, last practice collection date, transfer out of CPRD date, and GP registration end date.

In both SAIL and DataLoch, patient follow-up start date was minimum GP registration start date, while the follow-up end date was set to the earliest of: date of death, maximum GP registration end date and date of data extraction.

### Region

In CPRD, each practice had a region that corresponded to one of the nine International Territorial Level (ITL) 1 statistical regions of England (North East, North West, Yorkshire and The Humber, East Midlands, West Midlands, East of England, London, South East, and South West), plus Wales, Scotland, and Northern Ireland. As we only wanted to include patients in England, we dropped patients from practices with missing region or regions outside of the nine English regions. In SAIL, the entire cohort was assigned to Wales, and all patients in DataLoch were assigned to the Lothian region of Scotland.

### Socio-Economic Status

In CPRD, patient-level IMD 2019 quintiles were provided for our cohorts, linked by CPRD through the patient postcode at the LSOA-level in England. In SAIL, the latest WIMD 2019 quintile record for each individual was used to assign socio-economic status to the cohort, which was derived from the most recent LSOA. In DataLoch, the most recent GP registration per person provided the latest known postcode per patient, which was linked to SIMD 2020 v2 quintiles through the publicly available PHS SIMD dataset.

### Ethnicity

To assign ethnicity to each individual in the CPRD and SAIL cohorts, we adapted a codelist and algorithm previously used by,^25^ shown in Figure S2. Ethnicity was taken from GP datasets in both CPRD and SAIL, with hospital records additionally used to reduce missingness. In DataLoch, we used the internally derived ethnicity that was assigned to all patients within the database using a similar algorithm as used in these cohorts.

Note that the ethnicity codelist used incorporated many different categorisations of ethnicity in order to be flexible to research and region-specific needs. It included: England and Wales Census 2011 categories; Scotland Census 2011 categories; UK harmonised Census 2011 categories using Office for National Statistics guidance from^27^ and a broad six level ethnicity (White, Black, Asian, Mixed, Other).

### Smoking Status

All smoking status-related GP events in our cohorts were compiled to make a longitudinal smoking record per patient, in order to allow researchers to select the smoking status most relevant to their time period. Cleaning of the smoking status is described in Figure S3.

### Body Mass Index

Similarly to smoking status, we created a longitudinal BMI record for our cohorts by selecting all BMI-related GP events in both CPRD and SAIL.

All three data sources used the raw BMI values found in the GP records. However, to improve coverage of BMI events for SAIL and CPRD, we also cleaned raw weight and height values to derive additional BMI values, as has been performed in other CPRD studies (such as (20)) (Figure S4). In DataLoch, only the raw BMI values were used. More detailed information can be found in the supplement in the section I). All values were restricted to sensible ranges based on age and variable as seen in Table S1.

### Spirometry

For COPD, we created a longitudinal record of forced expiratory volume in one second (FEV1) in litres (L) and FEV1% predicted (%) values, restricted to the ranges presented in Table S2 based on previous published research such as,^28^ with additional columns denoting whether bronchodilation (if any) occurred before or after the spirometry test along with the Global Initiative for Obstructive Lung Disease (GOLD) staging of the value. Details of spirometry data cleaning can be found in Figure S5.

Where there was no GP-entered FEV1% predicted value on the same day as an FEV1 value for a patient, FEV1% predicted was derived instead. For every FEV1 value (highest value used where multiple values recorded on the same day), a predicted value was generated based on the patient’s age at measurement and sex, using the ERS (European Respiratory Society) ‘93 regression formulae seen in Table S3.^29^ Ethnicity is known to impact expected FEV1 hence these equations are recommended to be applied to people of White or White European origin (aged 18–70 years old), however these were applied to all patients for simplicity. The following formula was then implemented: FEV1% predicted (%) = FEV1 (L)/FEV1 predicted (L) * 100.

Given low levels of reported post-bronchodilation measurements (7.2% of all FEV percent predicted measurements in CPRD; 5.7% in SAIL; 0.2% in DataLoch), GOLD stage was pragmatically reported using all available FEV percent predicted measurements in line with previous research that excluding those with more questionable data can lead to selection bias.^28^

### ILD Classification

The ILD cohort was additionally given flags to indicate the presence of a code which indicated the type of ILD: IPF with both broad and narrow definitions as previously performed in^30^ using validated definitions^22^ (see https://github.com/NHLI-Respiratory-Epi/Validation-of-The-recording-of-Idiopathic-Pulmonary-Fibrosis-in-routinely-collected-electronic-healt/); exposure-related (ie hypersensitivity pneumonitis and pneumoconiosis); autoimmune-related (including sarcoidosis, rheumatoid arthritis-related and systemic sclerosis); treatment-related (ie drug or radiation-induced); and other. We chose not to give each individual a final classification as this can depend on the presence of non-ILD-related codes, which are likely more reliable from secondary care free-text clinical notes.

### Asthma/COPD Medications

For asthma and COPD treatments, we created a longitudinal record of commonly prescribed asthma/COPD medications from the GP prescription records in CPRD, SAIL and DataLoch. The categories of drugs included were: short-acting bronchodilators (SABA); short-acting muscarinic antagonists (SAMA); long-acting bronchodilators (LABA); long-acting muscarinic antagonists (LAMA); inhaled corticosteroids (ICS); SABA-SAMA; LABA-LAMA; LABA-ICS; oral corticosteroids (OCS); triple therapy; theophylline; Phosphodiesterase-4 (PDE4) inhibitors (COPD only) and antibiotics (COPD only).

As Read Codes DAAD were deprecated in 2016,^19^ newer drugs, including LABA-LAMA and triple therapy inhalers (Trelegy Ellipta^31^ and Trimbow^32^ both approved for use in UK in 2017), did not have assigned Read codes in SAIL meaning a different method of identifying these prescriptions was required. In SAIL, these medications required identification using their component drugs where they occurred on the same day. For example, a flag was established for triple therapy prescriptions where LAMA, LABA and ICS codes were all recorded on the same day.

## Results

We compared the asthma, COPD and ILD populations in CPRD, SAIL and DataLoch by key socio-demographic factors (age, sex, ethnicity, area-level deprivation, region (for England only)); patient characteristics (BMI, smoking status, spirometry (for COPD only), ILD classification, asthma/COPD medication usage) at the most recent time period included in the study—31st December 2019. This time point was selected to provide the most recent common endpoint across the data snapshots. For BMI, smoking status and spirometry, we used the most recent measurement available in the past five years, from 1st January 2015 to 31st December 2019 inclusive. For asthma and COPD medication usage, we looked at number of patients with any prescription in each drug category in 2019.

### Populations

#### Cprd

**Figure 1:**
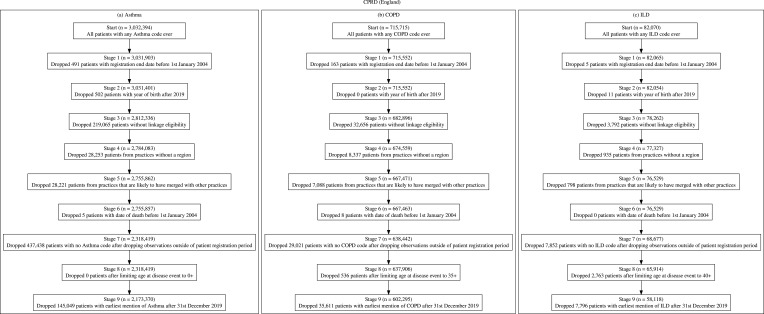
(**a**) CPRD asthma cohort inclusion and exclusion criteria with counts, (**b**) CPRD COPD cohort inclusion and exclusion criteria with counts, (**c**) CPRD ILD cohort inclusion and exclusion criteria with counts.

Following the minimisation of each cohort (see Figure 1a–c, respectively), the CPRD asthma cohort consisted of 2,173,379 patients, COPD cohort had 602,295 patients and ILD cohort consisted of 58,118 patients.

#### Sail

**Figure 2:**
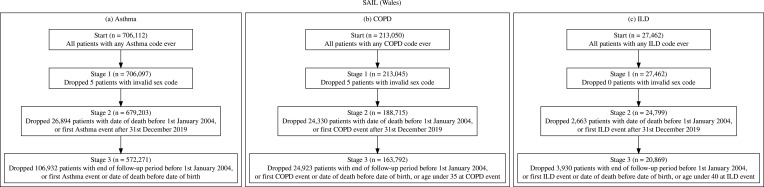
(**a**) SAIL asthma cohort inclusion and exclusion criteria with counts, (**b**) SAIL COPD cohort inclusion and exclusion criteria with counts, (**c**) SAIL ILD cohort inclusion and exclusion criteria with counts.

Following the cohort inclusion and exclusion criteria detailed in Figure 2a-c the SAIL the asthma cohort consisted of 572,271 patients, the COPD cohort had 163,792 patients and ILD cohort consisted of 20,869 patients.

#### DataLoch

**Figure 3:**
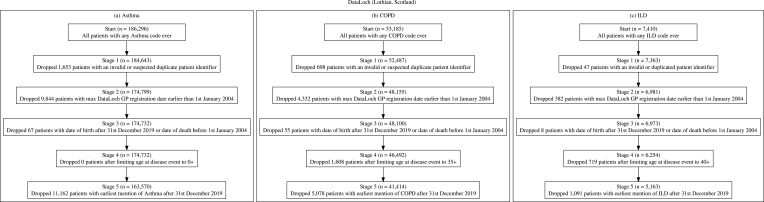
(**a**) DataLoch asthma cohort inclusion and exclusion criteria with counts, (**b**) DataLoch COPD cohort inclusion and exclusion criteria with counts, (**c**) DataLoch ILD cohort inclusion and exclusion criteria with counts.

After minimising each cohort (see Figure 3a–c, respectively), the DataLoch asthma cohort consisted of 163,570 patients, the COPD cohort contained 41,385 patients and ILD cohort consisted of 5160 patients.

### Comparison of Characteristics of People with Asthma, COPD and ILD in SAIL, CPRD and DataLoch

**Figure 4:**
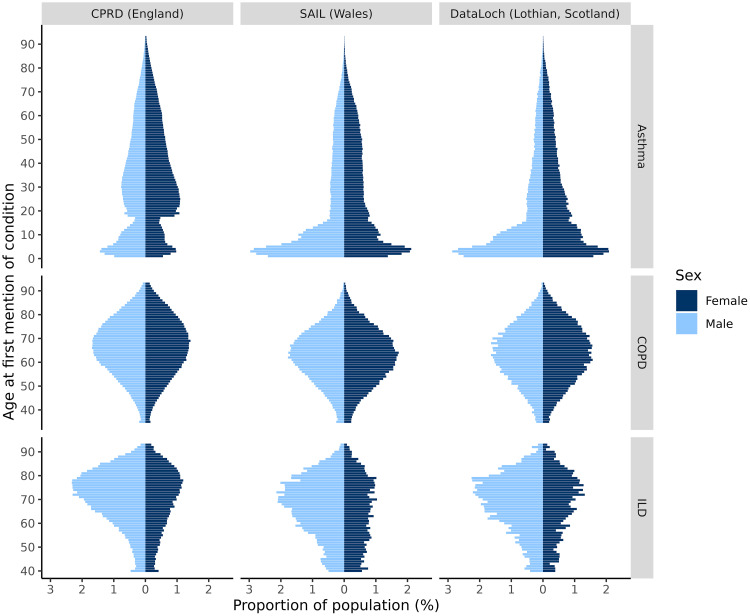
Proportion (%) of asthma, COPD and ILD populations by age at first mention of condition (ie earliest valid record with diagnosis) stratified by sex per data source: CPRD, SAIL and DataLoch. Age at first mention of condition limited to <=93 years across all EHR databases to minimise small numbers. The minimum age threshold for asthma, COPD and ILD are ages 1, 35 and 40, respectively.

The distribution of the asthma, COPD and ILD populations in each dataset by age at diagnosis (using age at earliest mention as proxy) and sex are shown in Figure 4. All three asthma cohorts had a clear peak in childhood (approximately 2–3 years old), however in CPRD the population had a strongly pronounced bimodal distribution, with another peak around 30 years old for males and 25 years old for females which may correlate with ages that people move home and change their practice. On the other hand, the COPD and ILD populations had similar distributions across all three datasets. The distribution of people by age of diagnosis was roughly symmetrical for COPD (around 60–70 years old) although with higher numbers of males overall, while for ILD in the three datasets the cohort population was dominated by older males (aged 70–80 years).

A comparison of characteristics of people ever diagnosed with asthma, COPD and ILD in CPRD, SAIL and DataLoch at the end of the observation period (31st December 2019) is shown in Tables S4–S6.

## Discussion

Through a harmonised approach to data curation, we have developed datasets on people with asthma, COPD, and ILD for the English, Welsh and Scottish populations including key demographic and clinical variables.

The data harmonisation approach allowed us to compare a range of key socio-demographic relationships with three chronic respiratory diseases—asthma, COPD and ILD—across three countries in the UK. Overall findings were similar across the datasets, for example consistent with the literature there were stronger sex differences in prevalence for ILD than COPD;^33^,^34^ IPF as the most prevalent type of ILD (1); and strong socio-economic gradients for COPD,^35^ but not for asthma and ILD. To date, there are relatively little published data on socio-economic status and ILD^36^ and the literature has been mixed for asthma.^37^,^38^

Interestingly, while the population distribution of age at earliest mention by sex was consistent across all three datasets for COPD and ILD, the distribution of asthma in CPRD was quite different in CPRD when compared to SAIL and DataLoch. In CPRD, there was a bi-modal distribution with peaks in both childhood and early adulthood while in SAIL and Dataloch the peak was in childhood and then incidence tapered off in adulthood. This difference may reflect a higher amount of prevalent data that are misconstrued as incident in CPRD due to lack of continuous follow-up of patients across different GP registrations. This may be more distinct for asthma than COPD and ILD given it has a substantially earlier average onset and therefore the population with asthma are more likely to move GPs post asthma diagnosis than the COPD and ILD populations.

Notably, there was much more variation in missingness for variables which relied on GP coding and more complex data management in extracting the data, such as ethnicity, smoking status, spirometry and BMI. The differing levels of missingness in these measures are likely influenced by usage of Read codes in SAIL and DataLoch compared with medcodeid/SNOMED-CT in CPRD Aurum (apart from the low ethnicity coverage in SAIL which is a known issue currently being addressed internally). Overall distributions were quite similar across all the datasets despite contrasting levels of completeness, particularly BMI where DataLoch had higher levels of missingness, yet the findings were not considerably different from CPRD and SAIL (where derived BMI values from weight and height were included).

As anticipated, missingness was higher for smoking status across all datasets for the asthma and ILD cohorts, compared with COPD where the main aetiological cause is smoking. However, for all conditions, there was a larger proportion of ex-smokers in CPRD, where there was less missingness, compared with SAIL and DataLoch. This highlights the high potential impact of missing data in EHR datasets.

Similarly, there was a much higher level of missingness in SAIL and DataLoch for FEV1% predicted values, likely due to the Read coding. The missingness in DataLoch was reduced from approximately 50% to 28.8% when secondary care spirometry data was included. Additionally, the inclusion of these data may explain why there was a higher proportion of GOLD stage 1 patients in DataLoch.

Harmonisation of medication data was particularly complex due to differences in the prescriptions data used within each database. We found a lower prevalence of some medications, noticeably LAMA, LABA-LAMA and triple therapy, prescribed in people living with COPD in 2019 in SAIL—which used deprecated Read codes—compared with CPRD and DataLoch, which utilised prodcodeid and BNF code, respectively. This was because several medications coded within CPRD did not have a corresponding Read code, likely because there were drugs approved after 2016 and/or the mapping from dm+d to Read through ATC was insufficient. Counts for LABA-LAMA and triple therapy were low in SAIL despite attempts to identify these combination therapies by matching component drugs prescribed on the same day. Prescription data in SAIL may therefore underestimate prevalence of drugs prescribed in Wales, particularly newer ones.

Additionally, the prevalence of the BNF-coded dispensed drugs in DataLoch was on average slightly lower than the prevalence of prescribed drugs in CPRD which is not surprising given these represent different measures of medication use (prescribed versus dispensed). In summary, caution should be taken when performing comparative analyses involving medication prescribing or usage across the three nations.

### Study Strengths and Limitations

We utilised three large representative datasets. The SAIL primary care dataset included 88% of the Welsh population (at the data extract date in March 2022), while CPRD covered 20% of the English population. The DataLoch dataset covered patients from 88% of Lothian GPs.

While we developed the cohorts for the datasets in unison, there were some high-level differences between the three datasets which may have impacted on the harmonisation process. Here, we have aimed to be transparent about these differences and how they were addressed in combining datasets. This means that the process used here can be replicated in the future, and where appropriate, data could be pooled improving precision.

The variables derived for the three cohorts were dependent on what is coded within GP and hospital records. These data were collected as part of routine clinical care rather than for research and there is variation in coding practices, which will affect the level of completeness and accuracy of data. While we have used harmonised codelists and algorithms across both datasets aimed at selecting the best estimates for use for research, the quality of the data is dependent on factors at the data collection level which cannot be completely resolved through data management. It is also possible that subtle differences in healthcare access, guidelines and respiratory quality improvement initiatives in each country may have influenced some of the disease recording and therefore differences in the cohorts seen.

There is also potential for selection bias related to patient-level health service use, as patients who use health services less frequently will be less likely to have codes for variables of interest. As the three conditions selected here represent serious chronic health problems, this is less likely to have affected results compared to the general patient population.

### Study Implications

Beyond the substantive epidemiological findings presented, we have created research-ready datasets harmonised across CPRD, SAIL, and DataLoch, which can now be utilised by other researchers studying these chronic respiratory conditions. The codelists generated by the project are publicly available in order to facilitate future research.

Future work utilising end-user experiences with the datasets is now needed to further develop the cohorts into the optimal form for this goal. As researchers use the cohorts, this may identify additional useful variables and their preferred format.

The harmonised data curation approach used in creating these cohorts could be adapted for other diseases, leading to increased standardisation or research practice in the use of EHRs as well as decreasing the time needed to generate datasets, so that more time can be spent on substantive analytical research.

### Conclusion

The rich data available from EHRs are a huge resource for health research; however, to utilise these effectively, harmonised approaches and sharing of resources and expertise in their use between the research community is key. Here, we have shared an approach to dataset generation with the aim of contributing to more effective health data research. We would encourage other researchers to adapt and develop our approach and share methodology to improve the efficiency and validity of research using EHRs for the future. We plan to extend this work to other important respiratory conditions.

## Data Sharing Statement

Data are available on request from the CPRD. Their provision requires the purchase of a license, and this license does not permit the authors to make them publicly available to all. Licenses are available from the CPRD (http://www.cprd.com): The Clinical Practice Research Datalink Group, The Medicines and Healthcare products Regulatory Agency, 10 South Colonnade, Canary Wharf, London E14 4PU. The Stata scripts used within CPRD have been made available via a project GitHub. Please contact the corresponding author to access.

For SAIL, researchers can apply to use the underlying data tables and the curation scripts on a collaborative basis within the SAIL trusted research environment, subject to the standard SAIL project application process and agreeing to a derived dataset policy (saildatabank.com/contact).

The DataLoch data are available as part of the DataLoch Respiratory Registry – a de-identified registry of linked respiratory data from the South-East Scotland region – which can be accessed by application to the DataLoch service (dataloch.org/connect-with-us).
